# The effects of continuous exposure to low-dose chlorine dioxide gas on the characteristics of induced pluripotent stem cells

**DOI:** 10.1016/j.reth.2022.07.014

**Published:** 2022-08-23

**Authors:** Ryoma Okawa, Koushirou Sogawa, Motoko Shiozaki, Kenji Yachiku, Takanori Miura, Takashi Shibata, Sachiko Ezoe

**Affiliations:** a Department of Space Infection Control, Graduate School of Medicine/Faculty of Medicine, Osaka University, 1-3 Yamadaoka, Suita, Osaka 565-0871, Japan; b Department of Clinical Pharmacy, Faculty of Pharmaceutical Sciences, Shonan University of Medical Sciences, 16-10, Kamishinano, Totsuka, Yokohama, Kanagawa 244-0806, Japan; c Strategic Global Partnership Cross-Innovation Initiative, Graduate School of Medicine, Osaka University, 2-2 Yamadaoka, Suita, Osaka 565-0871, Japan; d Department of Hematology and Oncology, Graduate School of Medicine, Osaka University, 2-2 Yamadaoka, Suita, Osaka, 565-0871, Japan

**Keywords:** iPS cells, Disinfectant, Low dose ClO_2_ gas, Stem cells, Cell fate

## Abstract

**Background:**

Recently, various regenerative therapies have been developed based on induced pluripotent stem (iPS) cells. However, hygienic control strategies have not been established at the manufacturing facilities. We aimed to evaluate the safety and effects of continuous exposure to low-dose chlorine dioxide (ClO_2_) gas on cell fates, and to determine the optimum dose for safe usage of this disinfectant.

**Methods:**

We cultured an iPS cell line in the absence or presence of various doses of ClO_2_ gas. We evaluated cell proliferation, cell death, the maintenance of undifferentiated state, and cell senescence.

**Results:**

We found that iPS cell proliferation was not affected by 0.05 or 0.1 ppmv ClO_2_ gas in the atmosphere. Although 0.1 ppmv ClO_2_ slightly affected apoptosis, it was not a significant effect. Moreover, neither at 0.05 nor 0.1 ppmv ClO_2_ gas significantly affected the characteristics of iPS cells.

**Discussion and conclusion:**

Continuous exposure to 0.05 or 0.1 ppmv ClO_2_ gas did not affect the fate of iPS cells. These results may contribute to the development of new strategies for hygiene control in cell processing facilities.

## Highlights

• IPS cells can be cultured in a low concentration of ClO_2_ gas without any effects on their fate.
• IPS cell proliferation and survival are not affected by 0.05 ppmv ClO_2_ gas in the atmosphere.
• IPS cell proliferation and senescence are not affected by 0.1 ppmv ClO_2_ gas in the atmosphere unlike in the case of MSCs.

## Abbreviations

Chlorine dioxide: ClO_2_
Cell processing facility: CPF
hydrogen peroxide: H_2_O_2_
cumulative population doubling level: CPDL
Mesenchymal stem cell: MSC
induced pluripotent stem: iPS

## 1 Introduction

Recently, the development and manufacturing of cell products for regenerative therapies, including immune-cell therapies, have accelerated. In particular, iPS cells, which were introduced in 2006, are now used in clinical treatments. These cells are induced from mature somaticcells with reprogramming technologies [1]. The development of regenerative medicine with iPS cells has promoted strategies for patients with retinal diseases, like age-related macular degeneration [2]; spinal cord injuries [3]; Parkinson's disease (PD) [4,5]; corneal diseases [[6], [7], [8]]; myocardial infarction [9,10]; diseases that cause thrombocytopenia, such as aplastic anemia and leukemia [11,12]; and genetic diseases, such as multiple sclerosis and recessive dystrophic epidermolysis bullosa [13].

Because regenerative therapies require healthy, living cells, the final products cannot be chemically or physically sterilized. Therefore, hygiene management should receive extra importance during the manufacturing process. In particular, for gene transplantations, viral vectors should be completely inactivated to avoid cross contamination.

To date, the hygienic environments of CPFs are mainly controlled by circulating air through high-efficiency particulate air filters. Thus, carried-in microbes are attenuated by air flow, but they cannot be perfectly removed. Accordingly, current research is focused on the most relevant approach for the disinfecting an entire space. In selecting and developing decontaminants for an entire space, emphasis should be placed on maintaining the safety of workers and avoiding the degradation of products and equipment, while achieving high sterilizing effects.

Although many types of agents have been used for decontamination in pharmaceutical plants and food factories, when selecting agents for CPFs, adverse effects on cell materials and on the working staff must be considered. In the cleanrooms of CPFs, the use of formaldehyde is currently restricted by the World Health Organization (WHO), due to its cancer-causing effects. Instead, hydrogen peroxide (H_2_O_2_), ClO_2_, ultraviolet (UV) light, and ozone are generally used for decontaminating the air in cleanroom environments. Among these decontaminants, from the viewpoints of both safety and effectiveness, we have focused on low concentrations of gaseous ClO_2_.

We previously reported that the characteristics of MSCs were affected little by continuous exposures to 0.05 ppmv ClO_2_ gas throughout long culture periods, but 0.1 mmv ClO_2_ caused MSC senescence, which inhibited proliferation [14]. The Environmental Protection Agency has set the maximum level of ClO_2_ in drinking water to 0.8 mg, and the Occupational Safety and Health Administration, an agency of the United States Department of Labor, has set the maximum permissible exposure limit for people working with ClO_2_ to 0.1 ppmv in air [14]. In previous studies, it was reported that 0.05 ppmv ClO_2_ gas, which is half of the 8-h time weight average concentration, had antibacterial and antiviral effects, and it inhibited the hyphal growth of fungi [15,16].

In this study, we examined the effects of continuous exposure to 0.05 ppmv ClO_2_ gas on the characteristics of iPS cell cultures.

## 2 Materials and methods

### 2.1 Cell culture

We purchased induced pluripotent human stem cells (409B2 human iPS cell line) from RIKEN BioResource Research Center (Ibaraki, Japan). 409B2 cells were maintained by single-cell passaging in 60 mm plates (Corning, NY, USA) coated with Easy iMatrix-511 (Takara Bio, Shiga, Japan). For passaging, 409B2 cells were harvested with EDTA/PBS (−) (Nacarai, Kyoto, Japan) diluted to 0.5 mM, and then replated at a density of 4000 cells/cm^2^. 409B2 cells were maintained for 22–24 h in StemFit AK02N medium (Takara Bio), which contained Y-27632 (Wako, Osaka, Japan), a Rock inhibitor. Next, the medium was changed to StemFit AK02N without Y-27632, and replenished every 2 days. The cells were passaged again on the 7th day. Then, the cells were divided into control and exposure groups. The control group was maintained in a CO_2_ gas incubator maintained at 37 °C. The ClO_2_ gas exposure group was maintained in a CO_2_ gas incubator continuously filled with ClO_2_ gas. We tested two different ClO_2_ concentrations: 0.05 ppmv and 0.1 ppmv.

### 2.2 ClO_2_ gas generation and measurement

ClO_2_ gas was stably generated in the culture incubator, according to a previously reported method [14]. The ClO_2_ gas concentration in the incubator was measured every minute with a dedicated gas analyzer (model GD-70D, Riken Keiki, Tokyo, Japan). The concentration error was within 30% throughout the experimental period.

### 2.3 Cell proliferation analysis

During each passage, a portion of the 409B2 cell suspension was mixed with an equal volume of 0.4% trypan blue solution (Sigma–Aldrich, MO, USA), and the number of viable cells was measured with an autocell counter (Model R1, Olympus, Tokyo, Japan). The CPDL (i.e., the number of times the cells doubled during the culture period) was calculated to assess the proliferative ability of 409B2 cells.

### 2.4 Analysis of differentiation potential

The expression of differentiation markers was assessed with flow cytometry on a fluorescence-activated cell sorter (FACS Canto II, BD Bioscience, CA, USA). Mouse monoclonal anti-human TRA-1-60, TRA-1-81, and SSEA-4 antibodies and a PE = conjugated mouse IgM kappa isotype control were purchased from BD Pharmingen (NJ, USA). Briefly, a 409B2 cell suspension was washed with PBS, then 500,000 cells were resuspended in 200 mL of FACS buffer, which included 1% FBS, and each antibody was added, respectively. These samples were then measured with flow cytometry, and data were analyzed with FlowJo™ Software Ver. 10 (BD Biosciences).

### 2.5 Cell death analysis

The death of 409B2 cells was evaluated with the FITC Annexin V Apoptosis Detection Kit I (BD Pharmingen). Measurements were performed, according to the manufacturer's protocol, on a FACS Canto II (BD Biosciences). FITC-conjugated annexin V and propidium iodide (PI) were detected at excitation/emission wavelengths of 488 nm/518 nm, and 535 nm/617 nm, respectively.

### 2.6 Evaluation of cellular senescence

The senescence of 409B2 cells was evaluated based on the expression of senescence-associated β-galactosidase (SA-β-gal). SA-β-gal activity was analyzed with the Cellular Senescence Assay Kit (CELL BIOLABS, CA, USA), according to the manufacturer's guidelines. Cell morphology and cell staining were observed with phase-contrast light microscopy.

We also evaluated cellular senescence in a quantitative manner, by assaying SA-β-gal with the fluorometric Quantitative Cellular Senescence Assay Kit (CELL BIOLABS), in accordance with the manufacturer's protocol. Briefly, cells were cultured with pretreatment solution at 37 °C for 2 h. Then, a SA-β-Gal Substrate Solution was added, and cells were incubated for 4 h or more. Finally, the cells were washed and separated, then subjected to flow cytometry analysis (excitation/emission: 488 nm/518 nm).

### 2.7 Quantitative real-time PCR

The analysis of 409B2 cell gene expression was performed with a Step One Real-Time PCR System (Thermo Fisher Scientific, MA, USA). Briefly, total RNA was extracted from 409B2 cells with the RNeasy Mini Kit (QIAGEN, Hilden, Germany). Then, RNA was reverse-transcribed to cDNA with SuperScript® IV Reverse Transcriptase (Thermo Fisher Scientific), and each target gene was amplified with PCR. The amplified products were detected with PowerUp™ SYBR® Green Master Mix (Thermo Fisher Scientific). Relative gene expression was evaluated with the 2^−ΔΔCt^ method. The sequences of each primer set (Takara Bio) are shown in supplemental Table 1.

### 2.8 Data analysis

All data are expressed as the mean ± standard deviation of 3–9 independent experiments. The obtained data were statistically analyzed with the Student's t-test. p < 0.05 was considered significantly different.

## 3 Results

### 3.1 Effects of continuous exposure to low-dose ClO_2_ on iPS cell proliferation

**Fig. 1:**
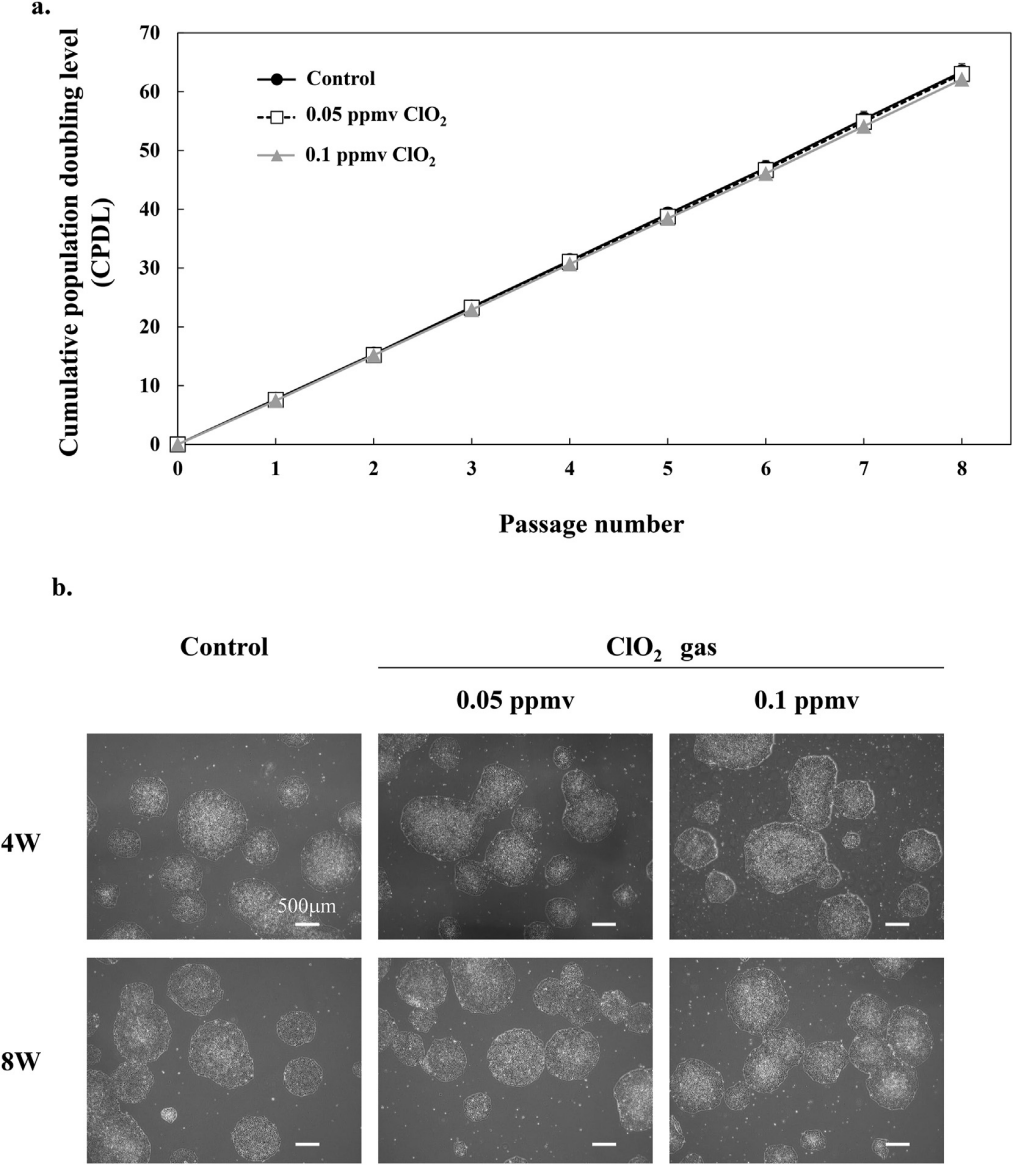
**Effects of ClO_2_ gas on 409B2 cell proliferation.** a. CPDLs of 409B2 cells cultivated for 8 weeks in various ClO_2_ gas conditions; *black circles*: 0 ppmv (control, n = 9), *white squares*: 0.05 ppmv (n = 3), *gray triangles*: 0.1 ppmv (n = 6). Data are expressed as the mean ± standard deviation. **b.** Light microscopy images of 409B2 cells at 4 weeks (upper panels) and 8 weeks (lower panels) after starting cultures without (control) or with 0.05 ppmv or 0.1 ppmv ClO_2_ gas. Scale bars: 500 μm.

To examine the effects of long-term ClO_2_ gas exposure on iPS cell proliferation, we cultured iPS 409B2 cells in a CO_2_ incubator in the presence or absence of ClO_2_ gas for 8 passages. We measured CPDL at each passage (Fig. 1a). The CPDLs of 409B2 cells cultured in the presence of 0.05 ppmv or 0.1 ppmv ClO_2_ gas were nearly the same as the CPDL of control cells. At the 4th and 8th passages (Fig. 1b), we found no differences between groups in morphological features, cellular density, or adhesion strength.

### 3.2 Effects of low-dose ClO_2_ on expression of proliferation-associated factors in iPS cells

**Fig. 2:**
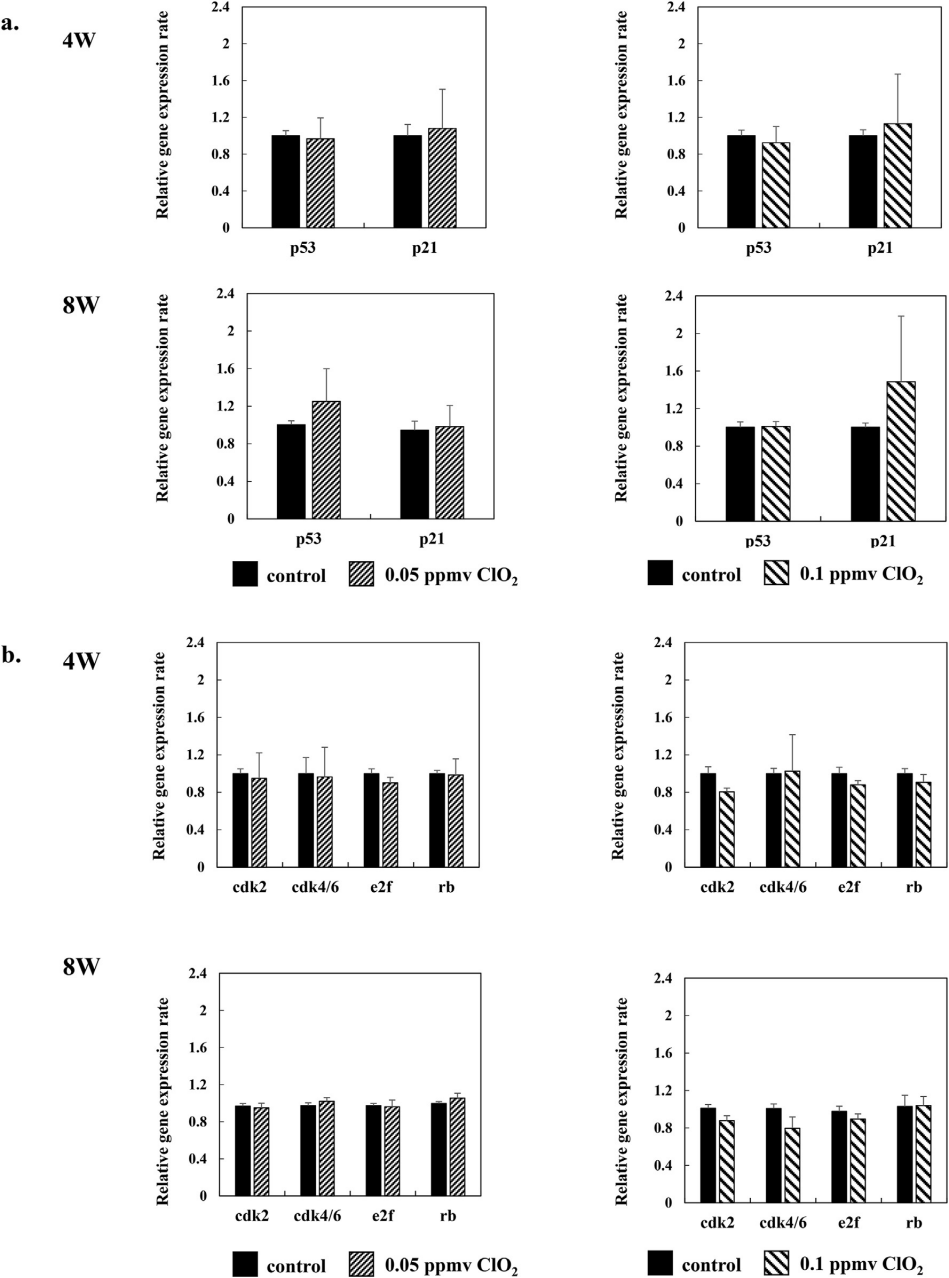
**Expression of cell growth-related genes in 409B2 cells exposed to ClO_2_ gas.** PCR results show: **a.** the relative expression of cell growth-inhibiting genes, p53 and p21, and **b.** cell proliferative factors, cdk 2, cdk 4/6, e2f, and rb, in 409B2 cells cultivated in the absence (control) or presence of 0.05 ppmv (*left panels*) or 0.1 ppmv (*right panels*) ClO_2_ gas. PCR was performed at the 4th (4 W, *upper panels*) and 8th passages (8 W, *lower panels*); Data are the mean ± standard deviation (n = 6).

To determine the mechanism of cell proliferation, we first analyzed the expression of factors that inhibited proliferation. We found no significant differences in the expression levels of p53 or p21 in 409B2 cells cultured in either 0.05 ppmv or 0.1 ppmv ClO_2_ gas, compared to the expression levels observed in control cells (Fig. 2a), at both the 4th and 8th passages. Next, we examined the expression of cell-cycle related genes, cdk 2, cdk 4/6, e2f, and rb. In 409B2 cells cultured in 0.05 ppmv ClO_2_ gas, the expression level of each gene was not significantly different from the level observed in control cells, at both the 4th and 8th passages (Fig. 2B). On the other hand, in cells cultured in 0.1 ppmv ClO_2_ gas, cdk2 expression was reduced at the 4th passage and cdk2 and cdk4 expression levels were reduced at the 8th passage, compared to expression levels in control cells (Fig. 2b).

These data suggested that, although the CPDL of iPS cells was not significantly affected by either 0.05 or 0.1 ppmv ClO_2_ gas, the proliferation of cells cultured in 0.1 ppmv ClO_2_ gas may have been slightly affected through the expression of cell cycle-related factors. In contrast, 0.05 ppmv ClO_2_ gas was insufficient to affect cell proliferation.

### 3.3 Maintenance of undifferentiated iPS cells exposed to low-dose CO_2_ gas

**Fig. 3:**
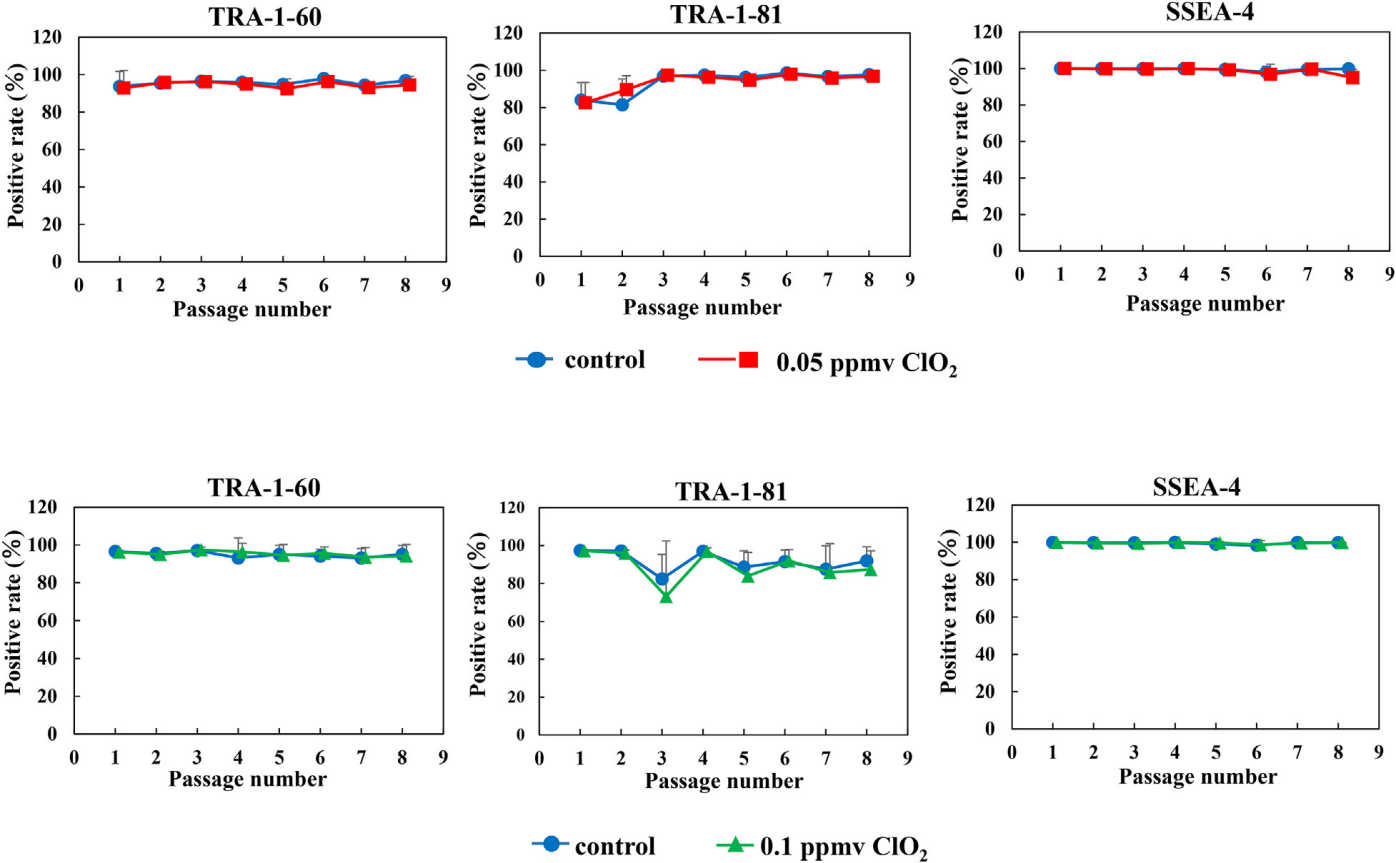
**Expression of surface differentiation markers in iPS 409B2 cells.** Line charts show the expression of surface markers for undifferentiated cells, TRA-1-60, TRA-1-81, and SSEA-4, from 1 to 8 weeks after starting cell cultures, in the absence (control) or presence of 0.05 ppmv (*upper panels*) or 0.1 ppmv (*lower panels*) ClO_2_ gas. Data are the mean ± standard deviation (n = 3). ∗p < 0.05 compared to control. *Blue circles*: control, *red squares*: 0.05 ppmv ClO_2_ gas, *green triangles*: 0.1 ppmv ClO_2_ gas.

Next, we examined whether ClO_2_ gas affected the undifferentiated state of 409B2 cells. TRA-1-60, TRA-1-81, and SSEA-4 are human stem cell-specific surface markers [17,18] that we evaluated with flow cytometry on the 8th day of passage. We found that nearly all the cells expressed these stem cell markers in each group (Fig. 3). When 409B2 cells were exposed to 0.05 or 0.1 ppmv ClO_2_ gas, the expression levels of these markers were not significantly different from the levels observed in control cells at any passage. From these results, we concluded that continuous exposure to ClO_2_ gas at either 0.05 ppmv or 0.1 ppmv did not affect the undifferentiated state of iPS cells, for at least eight passages.

### 3.4 Effects of low-dose ClO_2_ on cell death in iPS cells

**Fig. 4:**
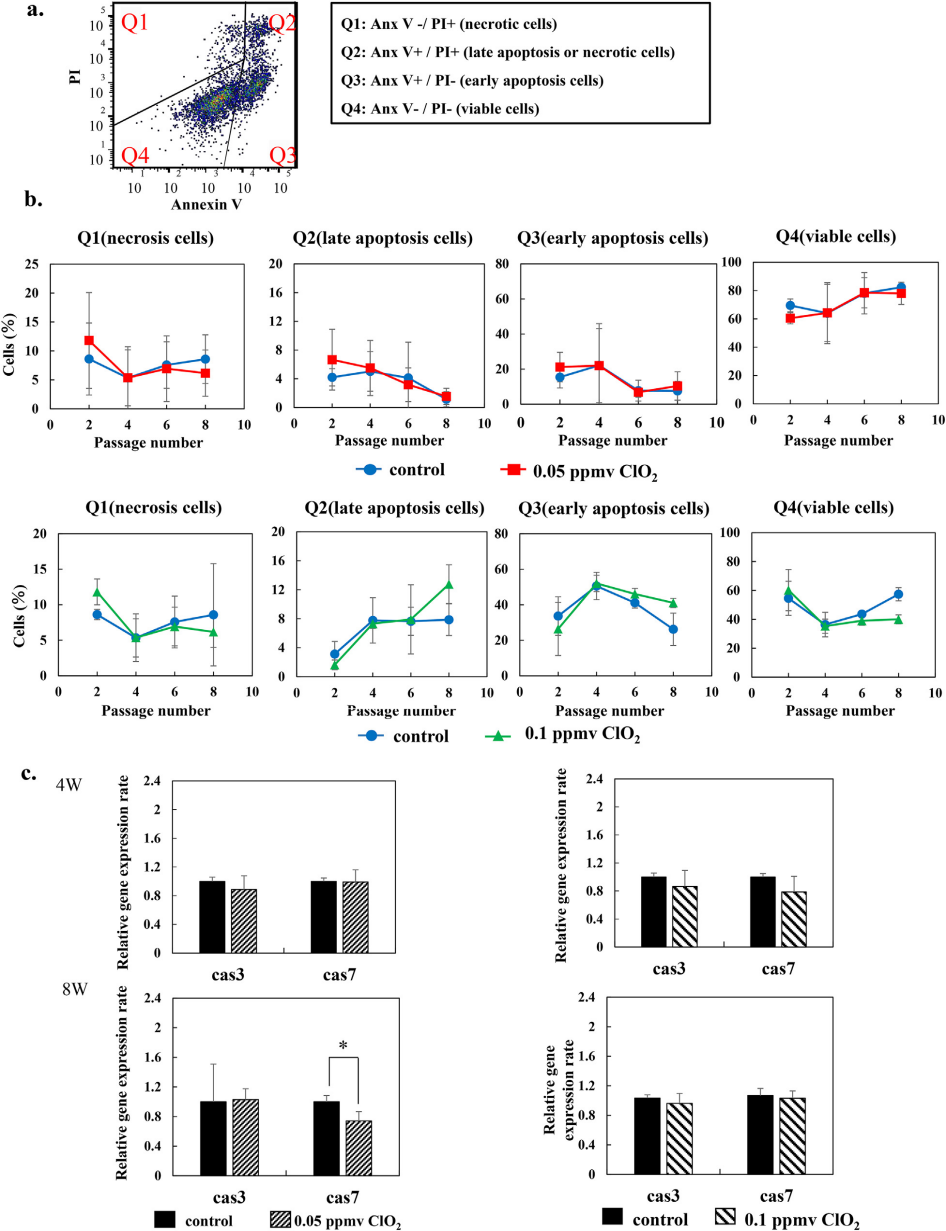
**Evaluation of cell death in iPS cells.** a. Flow cytometry separation of viable cells and cells that underwent different modes of cell death. Untreated 409B2 cells were cultured under normal conditions, stained with FITC-conjugated annexin V and PI, and subjected to flow cytometry. Q1: gated for necrotic cells, Q2: gated for late apoptosis or necrotic cells, Q3: gated for early apoptotic cells, and Q4: gated for viable cells. **b.** 409B2 cells were cultured for 8 weeks in the absence (control, *blue*) or presence of 0.05 (*red*, *upper panels*) or 0.1 ppmv (*green*, *lower panels*) ClO_2_ gas, and sub-cultured once per week. The percentages of cells detected in each gate for each passage are shown. **C.** RT-PCR results show the relative expression of cell death-related genes, cas3 and cas7, in 409B2 cells at weeks 4 and 8 (4 W and 8 W, respectively). All data are the mean ± standard deviation (n = 3). ∗p < 0.05 compared to control.

We investigated whether continuous exposure to low concentrations of ClO_2_ gas would induce cell death in 409B2 cells. Annexin-V was used to assess apoptosis, and PI was used to assess necrosis. Cell samples were analyzed with flow cytometry (Fig. 4a). Based on cell affinities for annexin V and PI, cells can be identified as viable cells (no affinity for annexin C or PI), early apoptotic cells (affinity for annexin C), late apoptotic/secondary necrotic cells (affinity for both annexin C and PI), and primary necrotic cells (affinity for PI). Fig. 4b shows how the abundances of these cell fractions changed over time. When treated with 0.05 ppmv ClO_2_, the abundance of each cell fraction was nearly the same as that observed in control cells, with no significant differences (Fig. 4b, upper panels). On the other hand, when 409B2 cells were cultured under 0.1 ppmv ClO_2_ gas, at week 8, the abundance of viable cells (Q4 fraction) decreased and the abundances of cells in early and late apoptotic stages slightly increased, compared to controls. However, these differences were not significant (Fig. 4b, lower panels). Furthermore, we examined the expression of apoptosis-related genes, Cas 3 and Cas 7 (Fig. 4c). Neither of these genes showed increased expression at week 4 or 8, when cells were treated with either 0.05 or 0.1 ppmv ClO_2_ gas, compared to control cells. Although, in some individual cultures, we observed a difference between the 0.1 ppmv and control groups, overall, neither 0.05 ppmv nor 0.1 ppmv ClO_2_ significantly affected cell death in iPS cells.

### 3.5 Effects of low-dose ClO_2_ exposure on cell senescence in iPS cells

**Fig. 5:**
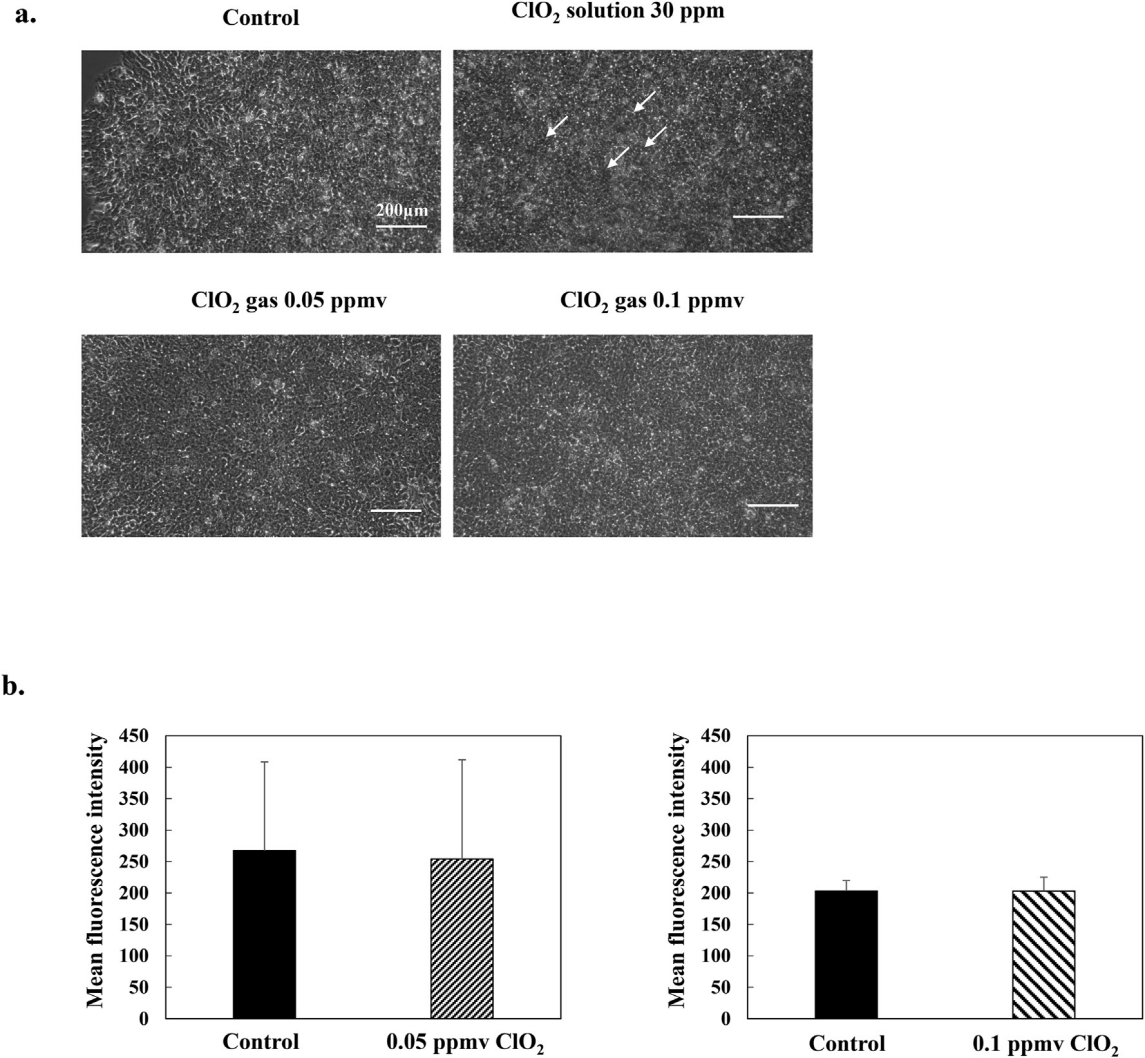
**Evaluation of cell senescence in iPS cells.** a. Images of 409B2 cells cultivated in the absence (control) or presence of ClO_2_ gas (30 ppm, 0.05 ppmv, and 0.1 ppmv) and stained with the cell-senescence marker, SA-β-gal (*white arrows*), at passage 8. The upper right panel shows the positive control cultured in the presence of a 30 ppm ClO2 solution. Scale bars: 200 μm. **b.** Cell senescence was evaluated quantitively with flow cytometry. SA-β-gal activity is shown as the mean fluorescence intensity. All data are the mean ± standard deviation (n = 3).

In a previous study, we demonstrated that 0.1 ppmv ClO_2_ gas, but not 0.05 ppmv, induced senescence in MSCs [18]. Here, we analyzed cell senescence by staining iPS cells cultured in ClO_2_ gas with SA-β-galactosidase. As shown in Fig. 5, few SA-β-galactosidase-stained cells were detected in the 0.05 ppmv, 0.1 ppmv, and control groups after 8 weeks of culture. These data, together with the data shown in Fig. 1a, suggested that, even exposures of 0.1 ppmv ClO_2_ had little effect on cell senescence in iPS cells, which was completely different from our observations with MSCs.

## 4 Discussion

In manufacturing regenerative therapy products, the final products cannot be sterilized; moreover, quality test results are sometimes not available until after administration to patients. Therefore, managing a sanitary environment is more important in manufacturing regenerative therapy products than in ordinary pharmaceutical operations. On the other hand, the raw materials, the intermediate materials, and the final end-products involve live cells, whose characteristics largely depend on the environmental conditions. In CPFs, the main approaches to hygiene control are to disinfect surfaces with alcohol or other disinfectants and to dilute internal dust with aerial filtration. In contrast, it is difficult to eliminate microbes, once imported into a CPF, because CPFs have intricate structures and equipment. In CPFs, the cell processing isolators and equipment are mainly sanitized by filling the entire space with hydrogen peroxide (H_2_O_2_) mist [19,20]. However, several previous studies have reported that decontaminating safety cabinets with H_2_O_2_ had effects on cell proliferation that persisted for long times [21].

Our research group has aimed to optimize a method for continuous disinfection in cell culture environments. We previously found that, under conditions of excess ClO_2_ concentrations, MSC proliferation was inhibited through the induction of senescence [14]. Recently, it has become possible to manufacture various types of iPS cells and related products [22]. However, iPS cell cultures require more careful control than MSC cultures. On the other hand, once epigenetic modifications have been cancelled, primitive iPS cells are highly proficient in antioxidant defense, similar to ES cells [23]. Although low levels of ROS are necessary for maintaining embryonic and adult stem cells [23,24], excess ROS levels initiate differentiation and can cause cell damage [[23], [24], [25]]. Here, we showed that, unlike MSCs which were inhibited by ClO_2_ gas [14], iPS cells did not exhibit cell senescence in response to the same dose of ClO_2_ gas. Moreover, iPS differentiation and proliferation were affected little. These results suggested that iPS cells are less susceptible to low oxidative stress than MSCs.

In conclusion, this study provided evidence that continuous exposure to ClO_2_, at concentrations with the least harmful effects to humans, but effective against microbes, had little effect on the characteristics of iPS cells. Additionally, we determined the optimum concentration for safe, effective decontamination of iPS cells. Future studies should examine the effects of ClO_2_ gas on differentiation potency to multilineage cells in long-term cultures. Establishing the safety of low-dose ClO_2_ gas for iPS cell cultures could initiate an epochal strategy in hygiene control for CPFs and research laboratories.

## Authorship contributions

RO performed experiments, collected and analyzed data, and wrote manuscript. KY conceived and designed the study, analyzed and interpreted data, and wrote the manuscript. MS, KY, and TM performed experiments and interpreted data. TS advised for design of experiments, supplied materials, and reviewed and approved the final draft of the manuscript. SE designed the study, interpreted data and wrote the manuscript. All authors reviewed the manuscript.
