# Supplementary material for: The effects of continuous exposure to low-dose chlorine dioxide gas on the characteristics of induced pluripotent stem cells
Source: Regen Ther. 2022 Aug 23;21:250–7. doi: 10.1016/j.reth.2022.07.014 (PMC9420961; doi:10.1016/j.reth.2022.07.014)
Supplement: Multimedia component 1 [file mmc1.pptx]

## Slide 1
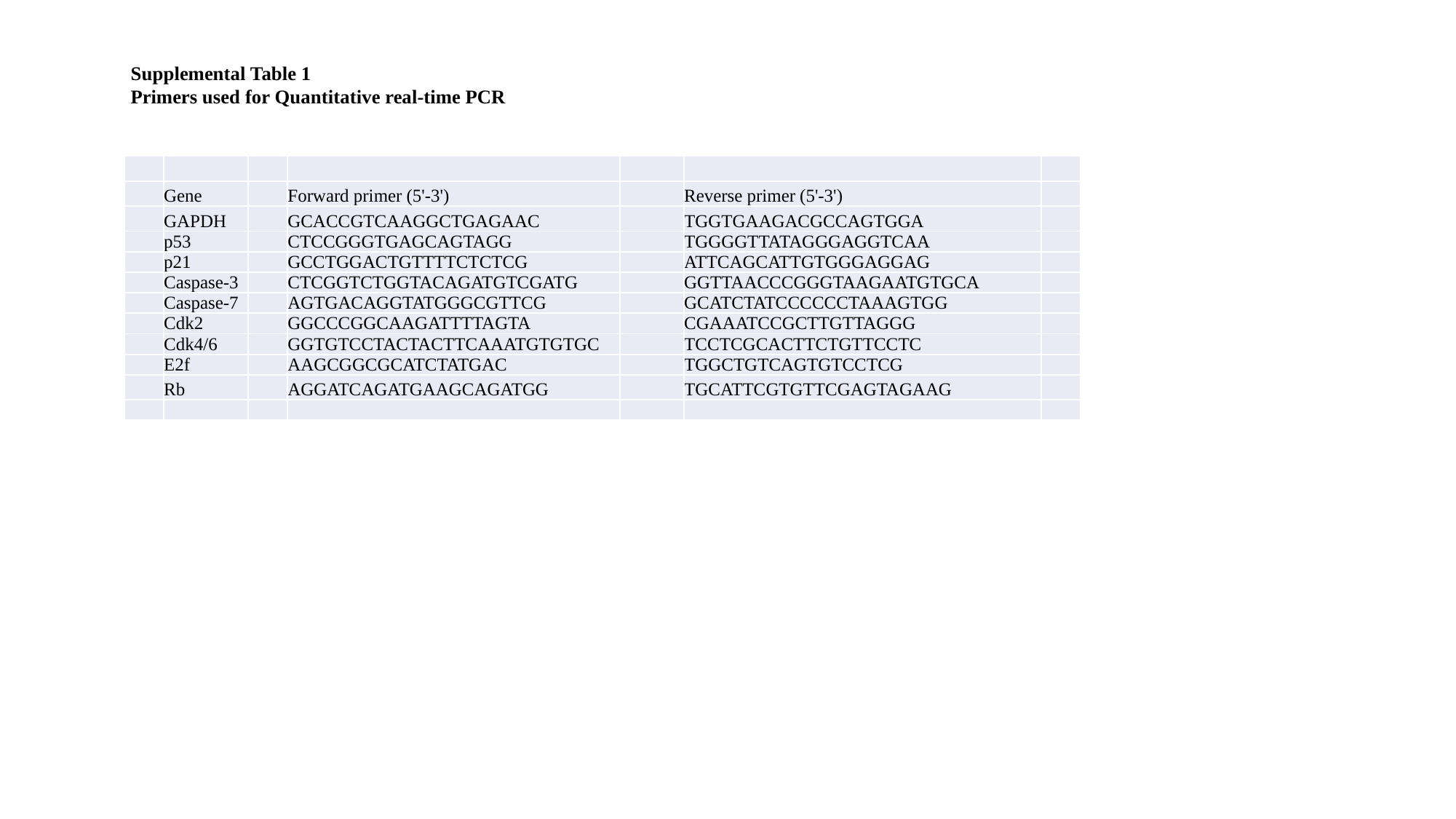

Supplemental Table 1
Primers used for Quantitative real-time PCR
| | | | | | | |
| --- | --- | --- | --- | --- | --- | --- |
| | Gene | | Forward primer (5'-3') | | Reverse primer (5'-3') | |
| | GAPDH | | GCACCGTCAAGGCTGAGAAC | | TGGTGAAGACGCCAGTGGA | |
| | p53 | | CTCCGGGTGAGCAGTAGG | | TGGGGTTATAGGGAGGTCAA | |
| | p21 | | GCCTGGACTGTTTTCTCTCG | | ATTCAGCATTGTGGGAGGAG | |
| | Caspase-3 | | CTCGGTCTGGTACAGATGTCGATG | | GGTTAACCCGGGTAAGAATGTGCA | |
| | Caspase-7 | | AGTGACAGGTATGGGCGTTCG | | GCATCTATCCCCCCTAAAGTGG | |
| | Cdk2 | | GGCCCGGCAAGATTTTAGTA | | CGAAATCCGCTTGTTAGGG | |
| | Cdk4/6 | | GGTGTCCTACTACTTCAAATGTGTGC | | TCCTCGCACTTCTGTTCCTC | |
| | E2f | | AAGCGGCGCATCTATGAC | | TGGCTGTCAGTGTCCTCG | |
| | Rb | | AGGATCAGATGAAGCAGATGG | | TGCATTCGTGTTCGAGTAGAAG | |
| | | | | | | |
